# Passion fruit plants alter the soil microbial community with continuous cropping and improve plant disease resistance by recruiting beneficial microorganisms

**DOI:** 10.1371/journal.pone.0281854

**Published:** 2023-02-21

**Authors:** Ye Wang, Yao Teng, Jianli Zhang, Zixiong Zhang, Chen Wang, Xiukun Wu, Xiuqin Long

**Affiliations:** 1 Guizhou Botanical Garden, Guizhou Academy of Sciences, Guiyang, Guizhou, China; 2 Institute of Mountain Resources of Guizhou Province, Guizhou Academy of Sciences, Guiyang, Guizhou, China; 3 College of Eco-environmental Engineering, Guizhou Minzu University, Guiyang, Guizhou, China; Sakarya Uygulamali Bilimler Universitesi, TURKEY

## Abstract

Passion fruit (*Passiflora edulis*) is widely grown in tropical and subtropical regions, showing high economic and ornamental value. Microorganisms are indicators for the stability and health of the soil ecosystem, which can affect the yield and quality of passion fruit under continuous cropping. High-throughput sequencing and interactive analysis were used to analyse the variation of microbial communities in the noncultivated soil (NCS), cultivated soil (CS), and the rhizosphere soil of purple passion fruit (*Passiflora edulis f*. *edulis* ×*Passiflora edulis f*. *flavicarpa*, RP) and yellow passion fruit (*Passiflora edulis f*. *flavicarpa*, RY). An average of 98,001 high-quality fungal internal transcribed spacer (ITS) sequences, mainly from *Ascomycota*, *Basidiomycota*, *Mortierellomycota*, *Mucoromycota* and *Glomeromycota*, as well as an average of 71,299 high-quality bacterial 16S rRNA sequences, mainly from *Proteobacteria*, *Actinobacteria*, *Acidobacteria*, *Firmicutes* and *Chloroflexi*, were obtained per sample. It was found that the continuous cropping of passion fruit increased the richness but reduced the diversity of soil fungi, while it dramatically increased the richness and diversity of soil bacteria. In addition, during the continuous cropping, grafting different scions in the same rootstock contributed to the aggregation of differential rhizosphere microbial communities. Among fungal genera, *Trichoderma* showed higher abundance in RY than in RP and CS, while the opposite was observed in the pathogen *Fusarium*. Moreover, the co-occurrence network and potential function analyses also showed that the appearance of *Trichoderma* was related to *Fusarium* and its contribution to plant metabolism was significantly greater in RY than in RP and CS. In conclusion, the rhizosphere of yellow passion fruit may be beneficial for the enrichment of disease-resistant microbes, such as *Trichoderma*, which may be an important factor inducing stronger resistance to stem rot. It will help to form a potential strategy for overcoming the pathogen-mediated obstacles in passion fruit and improve its yield and quality.

## Introduction

Passion fruit (*Passiflora edulis*), originating from South America, has been attractive to many consumers due to its bright colour, rich nutrients and unique flavour [1]. In recent years, the production area of passion fruit has doubled in China, surpassing Brazil by approximately 58.7% (IBGE, 2020). Two passion fruit cultivars, namely Tainong No. 1 and Fujian No. 3, cover approximately 95% of the planted area in China. Tainong No. 1 has a purple peel, intense flavour and higher cold tolerance, which is vulnerable to stem rot. Fujian No. 3, derived from an excellent single line of yellow fruits, has a yellow peel, higher sugar content and stronger disease resistance, but a milder flavour. The production scale of passion fruit in China has continued to expand, but viral diseases, stem rot, scab disease and brown spot, as well as high proportions of small and malformed fruits under continuous cropping strikingly reduce the yield and quality [2, 3]. With the increase of the market demand for passion fruit, continuous cropping has become a common practice, which usually leads to an obstacle in reducing plant growth and decreasing yield and quality by pests and diseases outbreak [4]. It is generally performed in grain and oil crops [5, 6], vegetables [7, 8], fruits [9, 10], herbal medicine [11, 12] and cash crops [13, 14]. In addition, continuous cropping has also been adopted for passion fruit in China [15].

According to preliminary investigations in different orchards, stem rot is one of the most important soil-borne disease of passion fruit and can destroy the whole garden when it breaks out. *Fusarium oxysporum* Schlecht and *Fusarium solani* (Mart) Sacc are pathogens causing stem rot of passion fruit in Guangdong, Guangxi, and Fujian provinces, and they mainly damage the base of plant stems in humid and hot environments, manifested as reddish-brown symptoms [16]. Due to a lack of effective fungicides, the diseases caused by *Fusarium* have always been a hard nut to crack. However, the large-scale planting of single cultivar and continuous cropping in orchards have aggravated the accumulation and spread of pathogens. In particular, there are few reports about the changes in soil microorganisms mediated by the continuous cropping of passion fruit.

Some studies have confirmed that the rhizosphere is the most critical part of the soil for nutrient exchange and information transmission, in which a unique root-soil-microbe microecosystem is formed [17]. Continuous cropping leads to an increase in pathogenic microorganisms in soil and disrupts the balance of the rhizosphere microecosystem [18, 19]. However, plants can change the components of metabolite secretions in light of different microbial species, which may not only enrich pathogenic bacteria but also increase beneficial antagonistic bacteria, triggering soil antibacterial effects [20]. In practice, yellow fruit with stronger disease resistance can be used as a rootstock to avoid the harm of *Fusarium* [21]. However, the mechanisms of different degrees of resistance between yellow fruit and purple fruit to *Fusarium* remain elusive.

Whether defensive microorganisms can be recruited to enhance the resistance of passion fruit to *Fusarium* is worthy of exploration. In this study, high-throughput sequencing and interactive analysis were adopted to analyse the structure of fungal and bacterial communities in noncultivated soil, cultivated soil and the rhizosphere soil of the two cultivars of passion fruit in continuous cropping, so as to provide new ideas for overcoming continuous cropping obstacles induced by *Fusarium*.

## Materials and methods

### Experimental site

The experiment was conducted in Guandong Town, Congjiang County, Guizhou Province, China (25°48′N, 109°01′E; 309 m above sea level), located in the subtropical monsoon climate zone. The average temperature and monthly precipitation during the growing season (from mid-March to mid-October) were 18.4°C and 149.8 mm, respectively. This typical yellow loam soil of the Yunnan-Guizhou Plateau exhibits the following characteristics: pH 4.52; organic matter content: 14.1 g/kg; total nitrogen content: 0.11%; effective phosphorus content: 25.7 mg/kg; available potassium content: 171 mg/kg; cation exchange capacity: 8.3 cmol/kg; and available nitrogen content: 168 mg/kg. The experimental plots were converted from a slope to a terrace and planted with passion fruit for five years.

### Materials and management

The materials include Tainong No. 1 (*Passiflora edulis f*. *edulis* × *Passiflora edulis f*. *flavicarpa*, referred as ’purple fruit’) and Fujian No. 3 (*Passiflora edulis f*. *flavicarpa*, referred as ’yellow fruit’), both of which were grafted onto the same rootstock. A randomized block design with three biological replicates was adopted. Thirty plants were planted in each plot (approximately 230 m^2^) with a row spacing of 3 m × 2.5 m. Ten kilogram of farm manure was applied to each plant, and a ridge mulching machine was used to open the boxes and ridges (surface width: 80 cm, bottom width: 100 cm and height: 35 cm). Large seedlings (plant height > 80 cm, new stem thickness > 0.4 cm and milky white young roots filling 1/3 of the culture cup) were planted in early April every year. Cement columns (3 m spacing) and iron wires were used to build a single-line fence frame for traction vines. Except for the noncultivated plots receiving zero management, the same agronomic management regime was applied throughout the growing season according to the local phenology. The experiment controled thrips, aphids, fruit flies and other insects by hanging yellow/blue sticky plates and releasing insect attractants, and removed insect eggs and shallow-rooted weeds such as *Poa annua* L. and *Alopecurus aequalis* Sobol. through soil tillage and exposure. The orchard was cleared at the end of December every year to prepare for replanting in the next year.

### Sample collection and processing

Soil samples were collected from noncultivated soil (NCS), cultivated soil (CS) subjected to the continuous cropping of passion fruit for five years and the rhizosphere soil of purple passion fruit (RP) and yellow passion fruit (RY) on October 14, 2020. Approximately 10–15 cm of topsoil was removed before sampling in the CS and NCS. According to the S-shaped five-point sampling method, soil sampling was performed at five points and mixed as one composite sample in each plot [22, 23]. Due to the smooth root surface and poor soil adhesion, the whole plants of RP and RY were dug up. The rhizosphere soil was obtained by gently brushing with a sterilized brush. Five plants were randomly selected and mixed as one sample in each plot. After the removal, mixing and filtering (10-mesh pore diameter) of residues, the samples were quickly placed on dry ice for deoxyribonucleic acid (DNA) extraction.

### Soil DNA extraction and sequencing

According to the instructions of the OMEGA Soil DNA Kit (D5625-01) (Omega Bio-Tek, Norcross, GA, USA), the total DNA was extracted from the soil samples. The concentration and purity of the DNA were detected by a NanoDrop ND-2000 spectrophotometer (NanoDrop Technologies, Wilmington, DE), and the DNA integrity was determined *via* agarose gel electrophoresis. Later, the DNA was uniformly diluted to 20 ng/μL. The internal transcribed spacer (ITS) region (primers: ITS-F 5’-GGAAGTAAAAGTCGTAACAAGG-3ʹ/ITS-R 5ʹ-GCTGCGTTCTTCATCGATGC-3ʹ) of fungi and V3-V4 regions of the 16S rRNA (primers: 16S-F 5ʹ-ACTCCTACGGGAGGCAGCA-3ʹ/16S-R 5ʹ-GGACTACHVGGGTWTCTAAT-3ʹ) of bacteria were used for polymerase chain reaction (PCR) amplification. A 7-bp specific barcode was integrated into the primers for multiple sequencing, and target fragments of 280 bp and 500 bp were obtained for the fungi and bacteria, respectively. PCR amplification was conducted in a 25 μL system consisting of 5 μL 5× reaction buffer, 5 μL of 5×GC buffer, 2 μL of dNTPs (2.5 mM), 1 μL of forward primer (10 μM), 1 μL of reverse primer (10 μM), 2 μL of DNA templates, 8.75 μL of ddH_2_O, and 0.25 μL of Q5 DNA polymerases. The thermocycler program was as follows: predenaturation at 98°C for 2 min; 30 cycles of denaturation at 98°C for 15 s, annealing at 55°C for 30 s and extension at 72°C for 30 s; and extension at 72°C for 5 min. After 1% agarose gel electrophoresis, the amplification product was purified with Vazyme VAHTSTM DNA Clean Beads (Vazyme, Nanjing, China), quantified with the Quant-iT PicoGreen dsDNA Assay Kit (Invitrogen, Carlsbad, CA, USA) and subjected to high-throughput sequencing on the Illumina-MiSeq platform (Shanghai Personal Biotechnology Co., Ltd). All sequences were deposited in the SRA database, which could be retrieved with Nos. SRP328580 and SRP328565 as keywords.

### Statistical analysis

According to the index and barcode information, the original sequence was preliminarily screened, and DADA2 from QIIME2 (https://view.qiime2.org) was used to perform primer removal, quality filtering, denoising, splicing, chimera removal and singleton removal. The results were clustered to obtain amplicon sequence variants (ASVs) at the 97% similarity level [24]. Then classify-sklearn classification metrics was used for ASV annotation in UNITE (release 8.0), Greengenes (release 13.8) and Silva (release132) databases [25]. Subsequently, the rarefaction method and the QIIME2 qiime feature-table rarefy function were employed for levelling (levelling depth: 95% of the minimum number of sample sequences) to obtain the specific composition of the microbial community at different classification levels [26]. At the genus level, the taxonomic grade tree of the top 100 genera was drawn in a circle packing [27].

Alpha diversity was analysed according to the Chao1 index, observed species, Shannon index, Simpson index, Pielou index and Good’s coverage, and the Kruskal-Wallis test was performed. The sample types were taken as a set, in which the ASVs were quantified, and the composition of the microbial communities among different sample types was visualized using a Venn diagram. In addition, a Jaccard and Bray-Curtis distance matrix and two-dimensional scatter plot of principal coordinate analysis (PCoA) were constructed using R software [28]. Finally, the unweighted pair-group method with arithmetic mean (UPGMA) and the stat package of R software were utilized for hierarchical clustering analysis.

Next, the correlation matrix was established with the SparCC algorithm according to random matrix theory (RMT). The first 100 genera showing average abundance were extracted to construct the co-occurrence network to identify the correlations among microbial components. Based on MetaCyc (https://metacyc.org/), KEGG (https://www.kegg.jp/) and COG (https://www.ncbi.nlm.nih.gov/COG/), PICRUSt2 was employed to predict potential functions [29]. The metabolic pathway abundance files and functional unit files were normalized according to the millionth of the total reference pathway of each sample. A statistical analysis of secondary metabolic pathways was performed using R software, and the map of the species composition contributing to different metabolic pathways was drawn at the genus level.

## Results

### Amplification, sequencing and annotation

The clear bands of 280 bp and 500 bp in length were obtained by PCR amplification for fungi and bacteria, respectively. The sequence alignment results showed that 1,377,723 sequences matched both forward and reverse primers by fungal ITS and reduced by 11.1% after removing the low-quality sequences. 1,208,311 effective sequences were obtained after denoising (an average of 100,693 per sample, ranging from 72,946 to 124,270) and 9,922 sequences were removed after splicing. 1,176,017 high-quality sequences were obtained after the removal of chimeras (an average of 98,001 per sample, ranging from 70,872 to 123,146), and three singletons were finally removed (S1 Table).

In addition, 1,228,744 16S rRNA sequences matched both forward and reverse primers of bacterial sequences and reduced by 6.8% after the removal of low-quality sequences. A total of 1,096,046 effective sequences were obtained after denoising (an average of 100,693 per sample, ranging from 74,092 to 110,121) and 146,716 sequences were removed after splicing. 855,593 high-quality sequences were obtained after the removal of chimeras (an average of 71,299 per sample, ranging from 53,946 to 90,976), and 20,169 singletons were finally removed.

**Fig 1:**
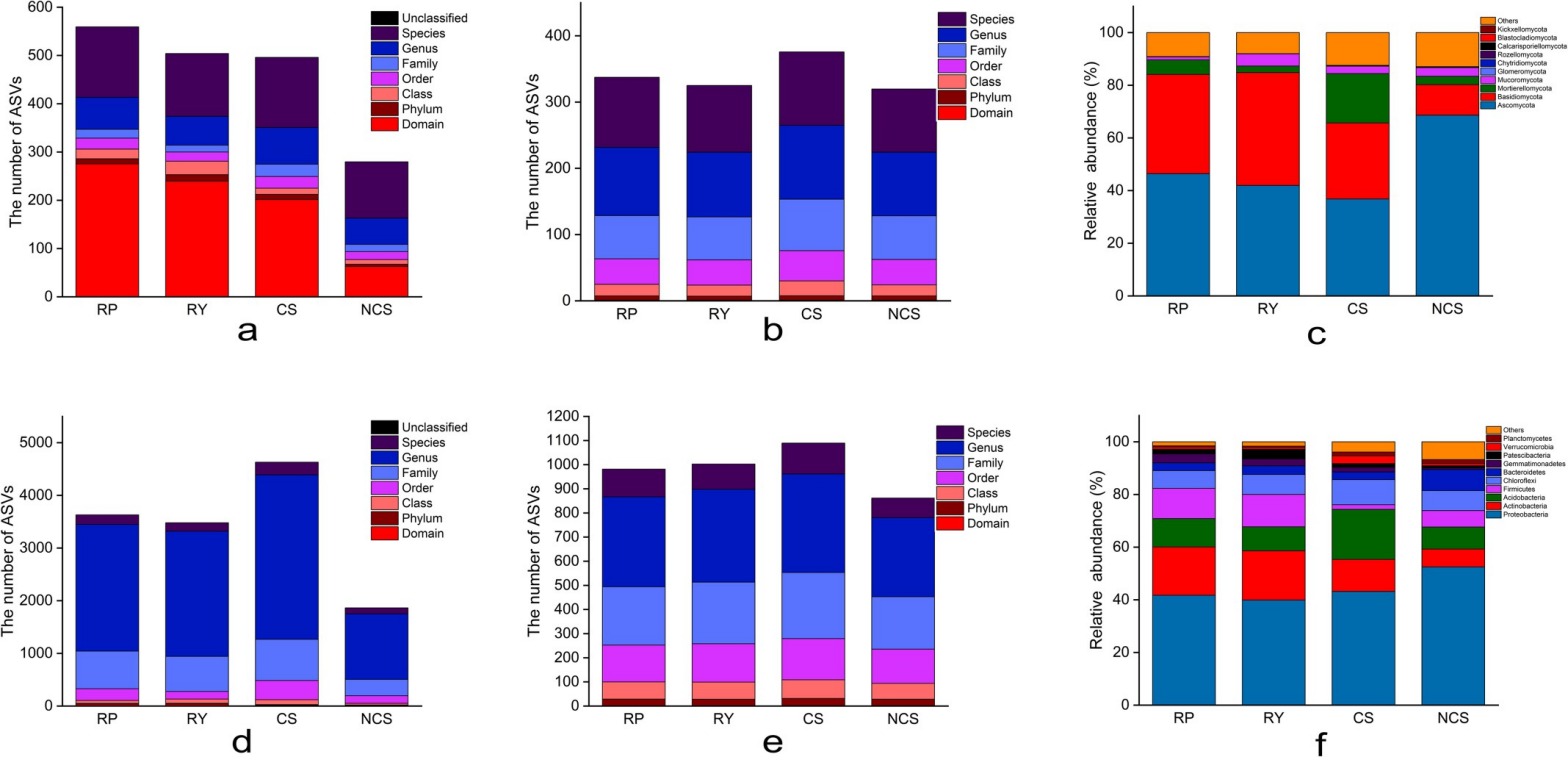
Annotation analysis of microbial composition in different soil and rhizosphere samples. (a, b, c) and (d, e, f) are the annotation analysis results of fungal and bacterial taxa respectively. (a, d) The number of ASVs in each sample that can be annotated to domains, phyla, classes, orders, families, genera and species. (b, e) The number of taxa contained in different samples at each classification level obtained from the flattened statistical data. (c, f) The abundance of the top 10 taxa at the phylum level. All data shown in the figure are averaged within the group.

The number of ASVs with ITS annotated to the domain level was significantly higher in CS, RP, and RY than in NCS (*p*<0.05, more than 3.4, 2.8 and 2.2 times, respectively), and that of ASVs with ITS annotated to the genus level was 66 (RP), 60 (RY), 76 (CS) and 54 (NCS), respectively (Fig 1A). Family and genus accounted for more than 19% and 29% of the taxa in all samples after denoising, removal and levelling, respectively (Fig 1B). Fungi dominating each sample included *Ascomycota*, *Basidiomycota*, *Mortierellomycota*, *Mucoromycota* and *Glomeromycota*, among which *Ascomycota* and *Basidiomycota* accounted for more than 65.7% of the total fungi (Fig 1C). The relative abundance of *Mortierellomycota* was significantly higher in CS than in RP, RY and NCS (S2 Table).

**Fig 2:**
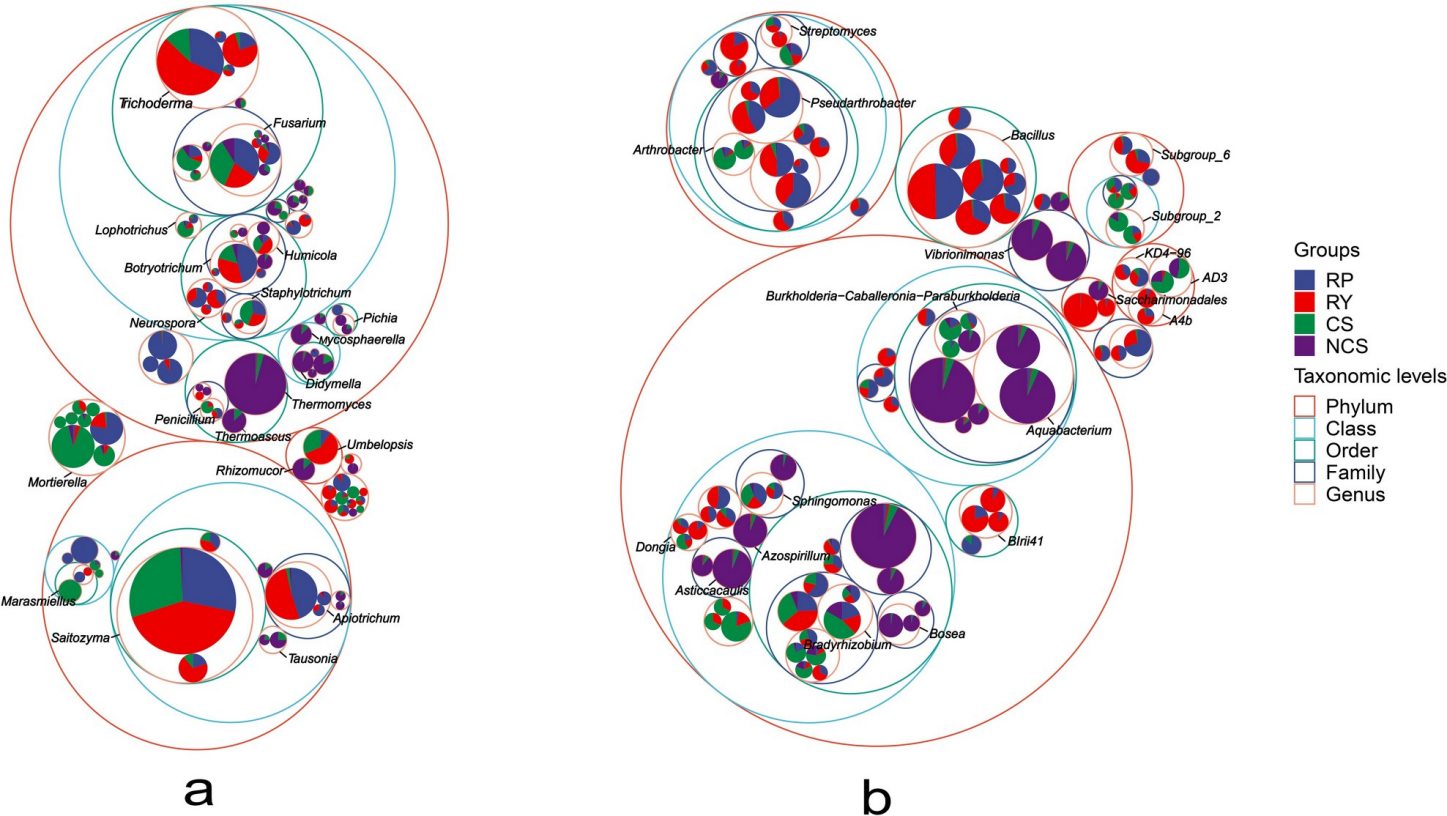
Circle packing of different soil and rhizosphere samples for fungi (a) and bacteria (b). The largest circle represents the phylum level, and the gradually shrinking circle represents class, order, family, and genus according to the gradient. The area of the fan in the circle represents the corresponding abundance and the top 20 genera have been identified with short lines.

However, the number of ASVs with 16S rRNA annotated to the family and genus levels in NCS was significantly lower than that in RP, RY and CS (Fig 1D and S3 Table), and annotated to the genus level was 1244 (NCS), 3125 (CS), 2408 (RP) and 2378 (RY), respectively, which accounted for more than 37% of the total sequences after levelling (Fig 1E). Among the top 10 phyla, the dominant bacteria in each sample belonged to *Proteobacteria*, *Actinobacteria*, *Acidobacteria*, *Firmicutes* and *Chloroflexi* (Fig 1F). The abundance of the bacteria in RP and RY showed consistent trends at the phylum level. The abundance of *Firmicutes* was significantly lower in CS than in RP, RY and NCS, and that of *Acidobacteria* was significantly higher in CS than in other samples (S4 Table). In this study, circle packing was used to visualize the overall composition of the microbial community. The results showed higher microbial abundance in RP, RY and CS than in NCS, and the abundance of bacteria was higher than that of fungi (Fig 2).

### Analysis of biodiversity

**Fig 3:**
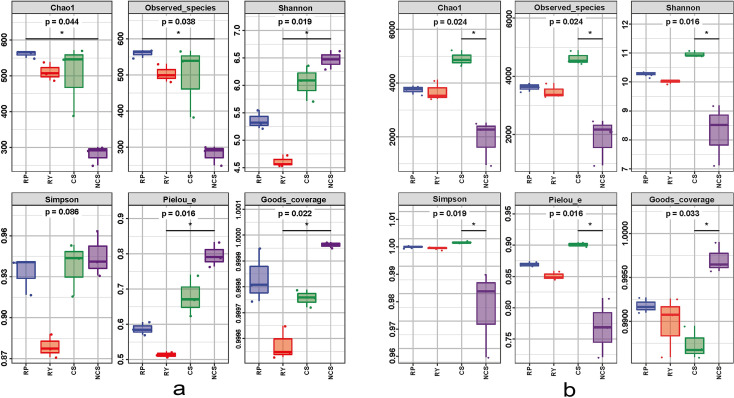
Alpha diversity analysis for fungi (a) and bacteria (b) in different soil and rhizosphere samples. The significance is expressed by the *p* value according to the Kruskal-Wallis test.

The Chao1 index and observed taxon values of the fungal and bacterial communities were significantly higher in CS than in NCS (Fig 3), which indicated that agricultural cultivation increased soil microbial abundance. Moreover, the Shannon and Simpson indexes were utilized to indicate the diversity of the microbial communities. The same trends were observed for these indexes in fungi (noncultivated > cultivated > rhizosphere), and only the Shannon index of NCS was significantly higher than that of RY. Regarding the bacterial sequences, the two indexes showed a different trend (cultivated > rhizosphere > noncultivated), and there was no significant difference in bacterial diversity between RP and RY samples. It appeared that continuous cropping increased the diversity of bacteria, and this impact was more remarkable than that on potentially attributable to the plant variety. The fungal uniformity in the rhizosphere was poor, and it was significantly lower in RY than in NCS according to the Pielou index. However, the bacterial uniformity was dramatically lower in NCS than in CS. The average coverages of fungi and bacteria were 99.98% and 99.15%, respectively, and the fungal coverage was notably lower in RY than in NCS, while the bacterial coverage was lower in CS than in NCS.

**Fig 4:**
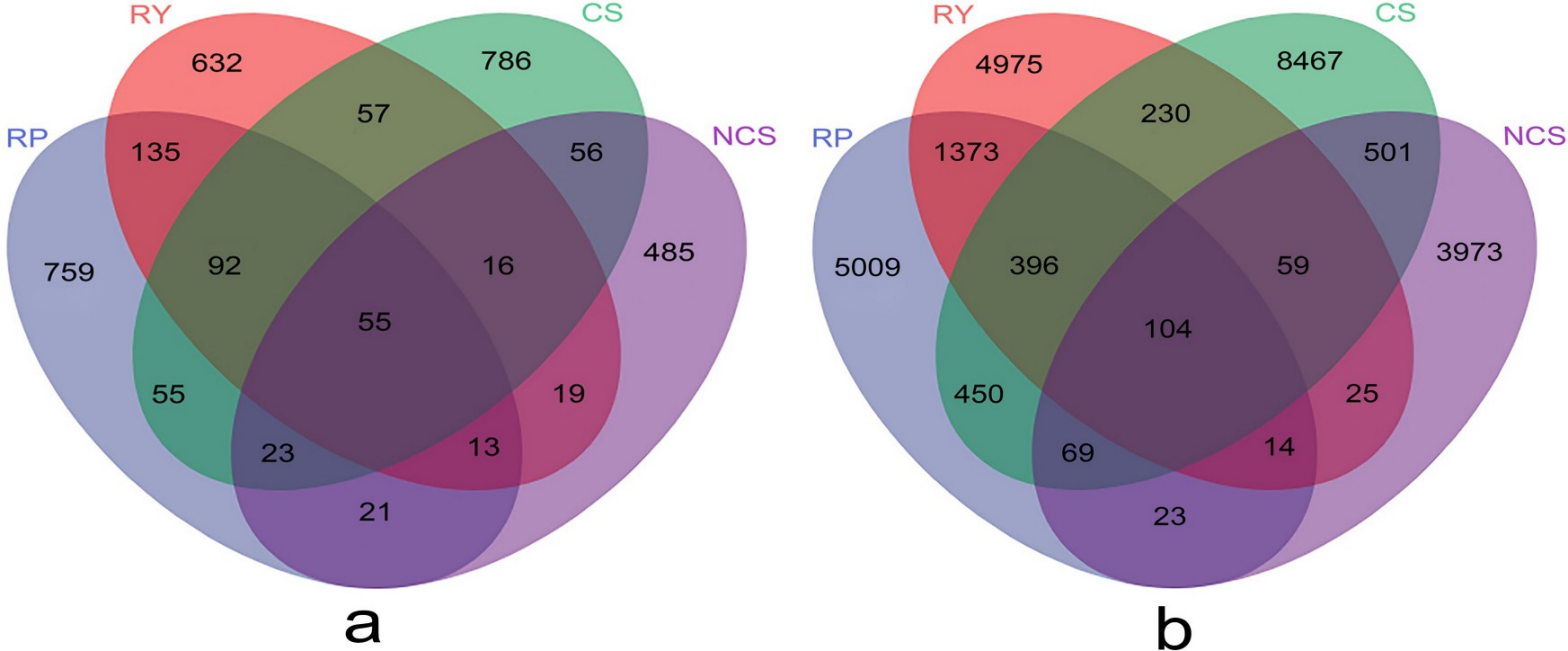
Venn diagram of unique and shared fungal (a) and bacterial (b) ASVs among different soil and rhizosphere samples. Each colour represents a group, and the numbers indicate the quantities of ASVs contained in the block.

The similarity and specificity of ASVs were visualized by a Venn diagram, which clarified the ASV distribution among the microbial communities in different samples. There were 759, 632, 786 and 485 fungal ASVs obtained from RP, RY, CS and NCS samples, respectively, among which 92 ASVs were found in RP, RY and CS samples, and only 55 ASVs were found in all samples (Fig 4A). Furthermore, 5009, 4975, 8467 and 3973 bacterial ASVs were obtained from RP, RY, CS and NCS samples, of which 396 ASVs were found in RP, RY and CS samples, and only 104 ASVs were found in all samples (Fig 4B).

**Fig 5:**
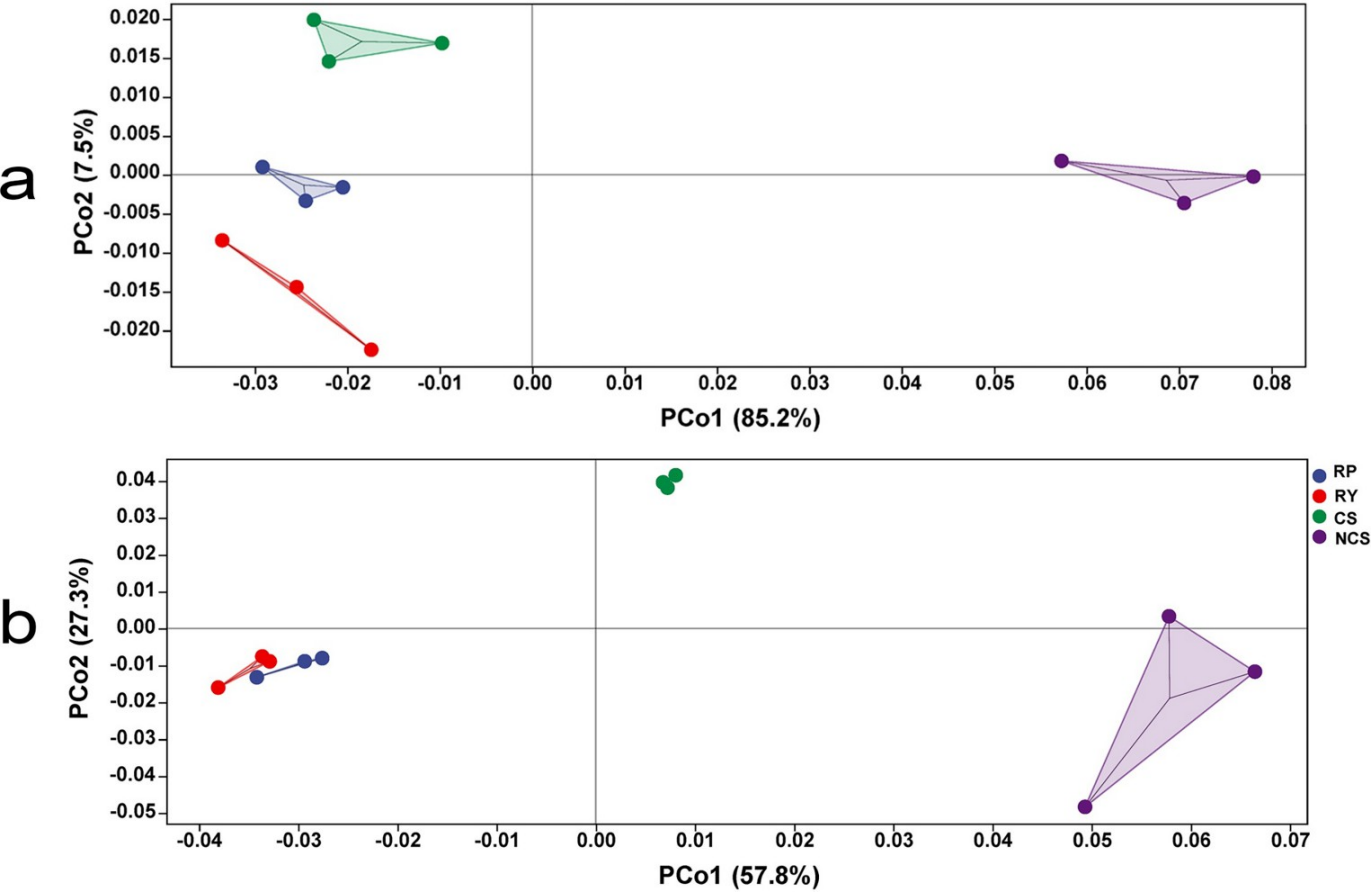
PCoA of fungi (a) and bacteria (b). Using the normalized functional unit abundance table (abundance sum of 1 M per sample), the distance matrix was calculated and PCoA analysis was performed by R Script. The percentage in the brackets on the axis represents the proportion of the sample variation that can be explained.

Based on the distance matrix and PCoA, PCo1 and PCo2 explained 85.2% and 7.5% of the sampling variation in the fungal data, respectively (Fig 5A). It should be noted that NCS made a greater contribution to PCo1 than other samples, while RP and NCS made only a small contribution to PCo2. In terms of bacteria, PCo1 and PCo2 explained 57.8% and 27.3% of the sampling variation in the bacterial data, respectively (Fig 5B). In general, RP and RY were close to each other and separated from CS and NCS. Among them, CS made the smallest contribution to PCo1 but the largest contribution to PCo2, while RP and RY were of the same low value in contribution to the two dimensions, especially to PCo2.

**Fig 6:**
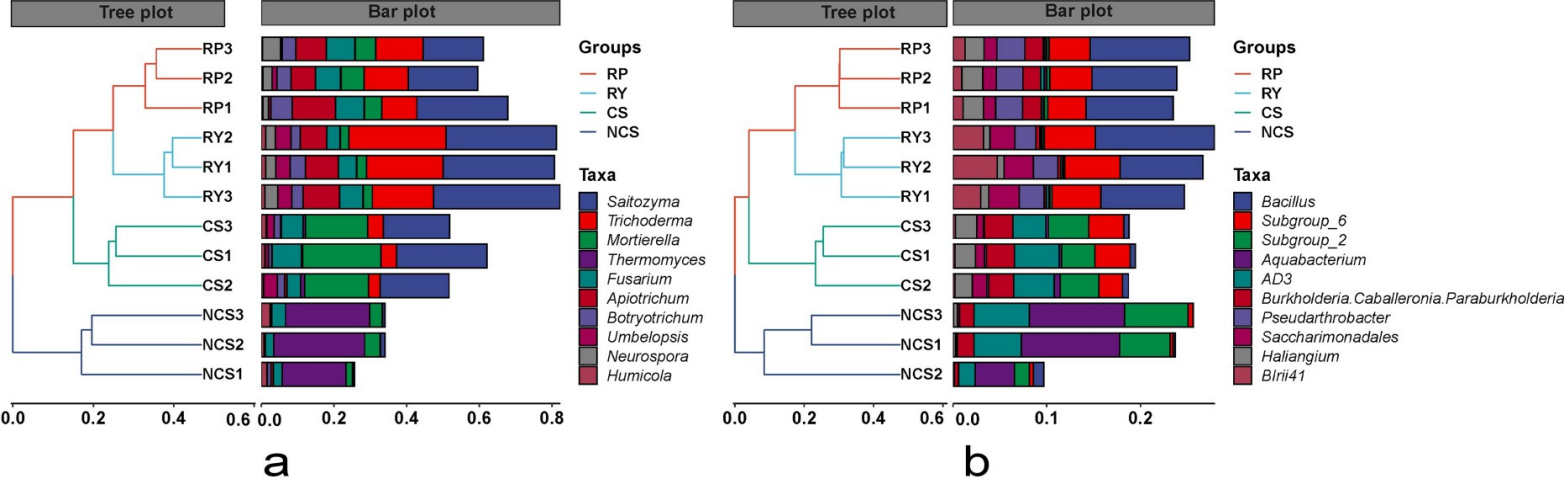
Hierarchical cluster analysis of fungi (a) and bacteria (b) in different soil and rhizosphere samples. The left diagram is a hierarchical clustering tree diagram according to the similarity between samples, and the right histogram is a stacked histogram of the top 10 genera. The phylogenetic trees were calculated using the neighbour-joining method.

The results of hierarchical cluster analysis showed that the abundance of *Trichoderma* was higher in RY than in RP, CS and NCS, while the abundance of *Mortierella* and *Fusarium* was lower in RY than in RP and CS. In addition, it was found that *Thermomyces* was abundant only in NCS (Fig 6A). The bacterial community structures in the rhizosphere and bulk soils were different. The accumulation of *subgroup*_*2* and *AD3* was significantly reduced, while that of *Bacillus* was significantly increased in the rhizosphere of passion fruit (Fig 6B and S5 Table). Notably, the genus *Burkholderia*, including the isolates of plant growth-promoting rhizobacteria (PGPR), showed significantly greater abundance in CS (*p* = 0.013) and RP (*p* = 0.005) than in RY.

### Co-occurrence network and potential function prediction

**Fig 7:**
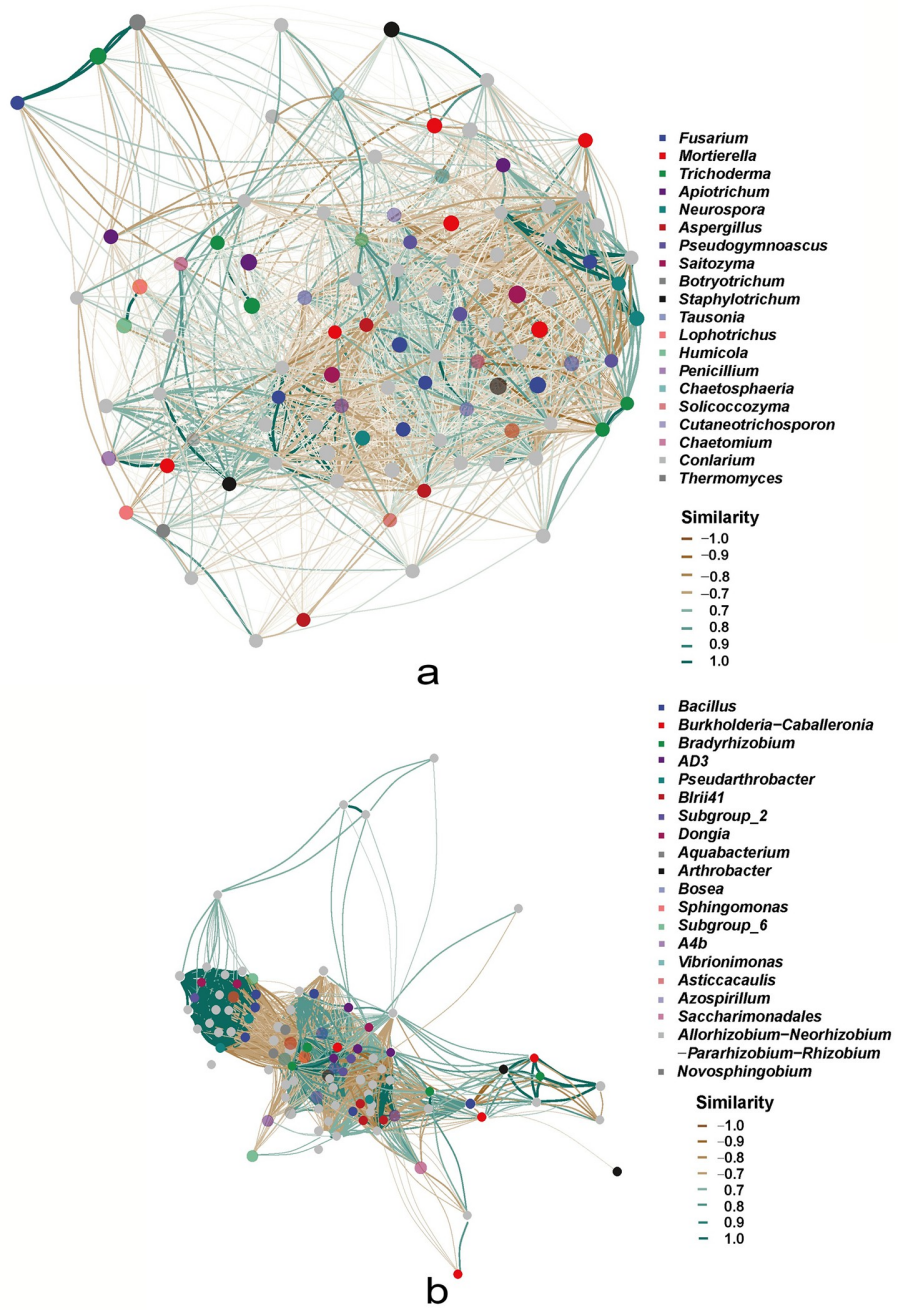
Co-occurrence network among the first 100 genera of fungi (a) and bacteria (b). The correlation matrix is constructed using the SparCC algorithm, but ASVs with a total number of sequences less than 10 are filtered based on random matrix theory (RMT) before matrix construction, with thresholds of 0.33 (fungi) and 0.6 (bacteria) (Chi-Square, *p*≤0.05). The top 100 ASVs were extracted to construct a network by the induced_subgraph function of igraph R package. The deeper and thicker lines represent stronger correlations between genera.

The co-occurrence network showed that *Trichoderma* exhibited significant correlations with *Fusarium and Botryotrichum* (Fig 7A). However, *Bradyrhizobium* was markedly related to *Burkholderia*, *Arthrobacter* and *Bosea* (Fig 7B). To clarify the potential functions of different taxa in the soil microenvironment, the abundance data corresponding to the main metabolic pathways from silico analyses of each sample were obtained by Minpath based on the KEGG, MetaCyc and COG databases. The relative abundances of fungi possibly involved in biosynthesis, degradation/utilization/assimilation, precursor metabolite/energy generation, glycan pathways and metabolic clusters were 25,046, 7,654, 19,460, 771 and 3,339, respectively (S1 Fig). RP, RY and CS showed 54, 52 and 31 pathways that were significantly different from NCS samples (S6–S8 Tables). Besides, there were 160,524, 40,992, 362, 38,450, 1,896, 1,589 and 6,185 bacterial sequences involved in biosynthesis, degradation/utilization/assimilation, detoxification, precursor metabolite/energy generation, glycan pathways, macromolecule modification and metabolic clusters, respectively. RP, RY and CS exhibited 190, 190 and 129 pathways that dramatically differed from those of the NCS samples, respectively (S9–S11 Tables).

**Fig 8:**
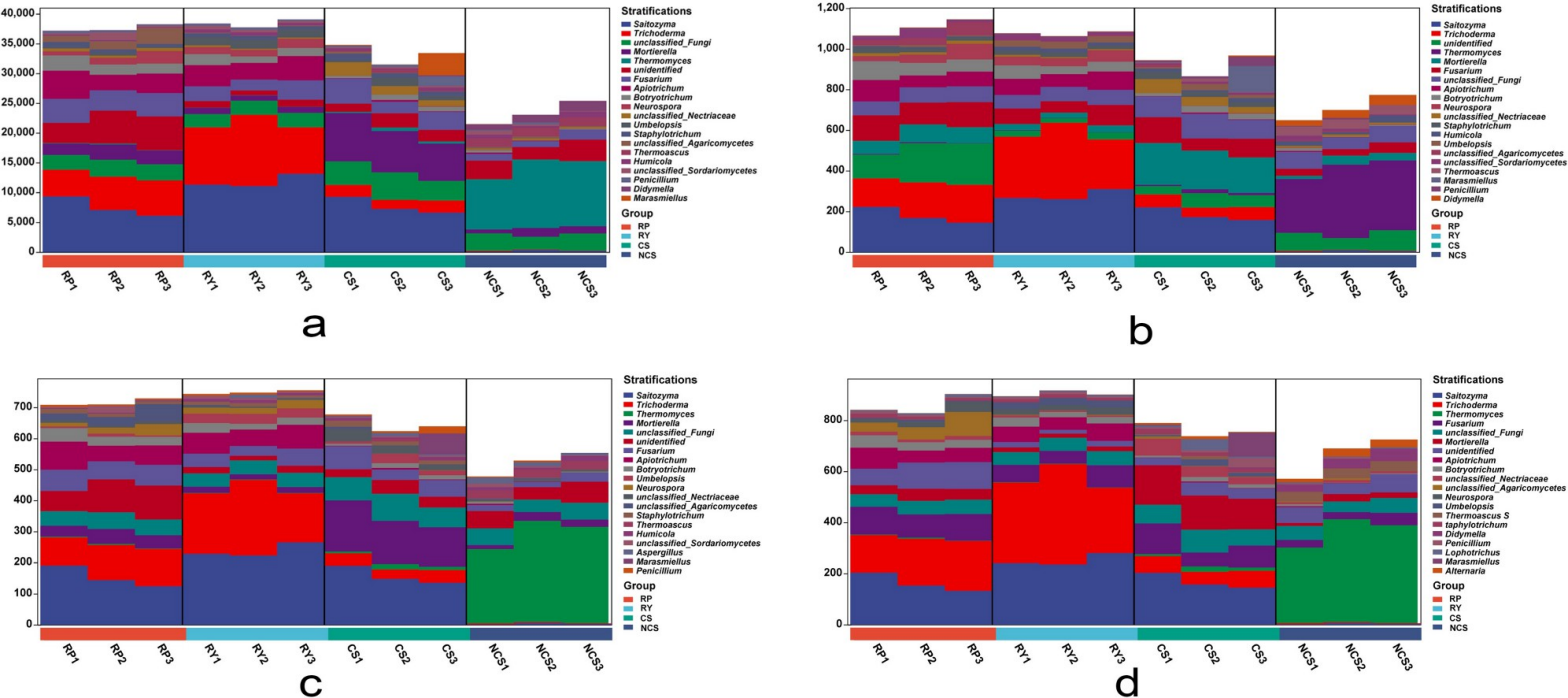
Contribution of fungal genera to different metabolic pathways. Different colours represent the contributions of different taxa to the glycolysis pathway (a), the glyoxylic acid pathway (b), the phosphopantothenic acid biosynthesis (c) and the non-oxidative pentose phosphate pathway (d) at the genus level (the first 20 genera).

In this study, we analysed the potential contributions of fungal genera to the glycolysis (anaglycolysis-PWY), the glyoxylate cycle (glyoxylate bypass), the nonoxidative pathway of pentose phosphorylation (nonoxipent-PWY) and the phosphopantothenic acid biosynthetic pathway (panto-PWY). The results showed that *Saitozyma* and *Trichoderma* greatly contributed to RP, RY and CS. In addition, *Saitozyma* made the greatest contributions to the glycolysis, the glyoxylic acid cycle and the phosphopantothenic acid biosynthesis pathway (relative abundance 16.24–34.11%), while *Trichoderma* made the greatest contribution to the non-oxidative pentose phosphate pathway (relative abundance: 20.37–35.56%) (Fig 8). Notably, the average metabolic contribution of *Trichoderma* in RY was significantly higher than that in RP (*p* = 0.026) and CS (*p* = 0.003), indicating that *Trichoderma* may tend to differentially accumulate in the rhizosphere of yellow passion fruit under the same conditions.

## Discussion

This study was designed to explore whether continuous cropping can affect the soil microbial diversity in passion fruit and to determine the difference in the rhizosphere microbes associated to the stem rot susceptible-cultivar (purple passion fruit) and resistant-cultivar (yellow passion fruit). The richness and diversity of soil bacterial and fungal communities were altered by the cultivation and continuous cropping of passion fruit. At the same time, the rhizosphere of the two cultivars was differentially enriched in the diverse genera of microorganisms to form a unique microbial structure. In China, passion fruit is mainly planted in areas showing a longer hot and rainy season, such as Guangxi Province, Fujian Province and Yunnan Province, where high temperature and high humidity create favourable conditions for the occurrence of fungi-mediated stem rot. In this study, more attention was given to fungi that exhibit great destructive effects on the production of passion fruit.

The soil microbes play an extremely important role in the formation and decomposition of organic matter, the circulation of soil nutrient elements, the plant growth and development, and the prevention of diseases and insect pests. They are affected by land use, cultivation history, agricultural management, tillage systems and changes in soil’s physical and chemical properties, such as pH and salinity [30–33]. With the increase of the duration of continuous cropping of passion fruit, the soil pH declines, and soil microorganisms transform from bacterial dominance to fungal dominance, which is more conducive to the reproduction and infection of soil pathogens, thereby affecting the growth of passion fruit [34]. As confirmed by previous research, the soils of passion fruit plants under longer continuous cropping have negative effects on the replanted of passion fruit seedlings, suggesting the presence of soil auto-toxicity in passion fruit [15].

Some microbial properties can be used to predict changes in the soil, providing an integrated and relevant vision of soil function [35]. Most strains are not culturable, which is the biggest challenge for prediction, but such a problem can be approached through the use of in-silico analyses based on the big data of high-throughput sequencing. In this study, the continuous cropping effectively increased the richness of soil fungi but reduced their diversity (Fig 3), which may pose selective stress on pathogenic and beneficial fungi and inhibit the development of the fungal community structure [36]. The sum of the relative abundances of *Saitozyma*, *Mortierella*, *Trichoderma* and *Fusarium* was greater than 70% (Fig 6). In particular, the genus *Saitozyma* dominated the microorganism community in CS, RY and RP samples, where it showed a relative abundance greater than 30%, consistent with the succession of forest soil microorganisms [37]. *Mortierella* is a valuable decomposer in agricultural soils and plant tissues, and some strains of this genus belong to the plant growth-promoting fungi (PGPF), which contribute to higher resistance to soil-borne pathogens or improves plant growth by dissolving organic C and available N, increasing chlorophyll content, and boosting the synthesis of total phenolics, flavonoids, carotenoids (apocarotenoids) and jasmonic acid (JA) [38–40]. In this study, *Mortierella* was detected in the bulk soil, cultivated soil and rhizosphere soil, and its proportion was the highest in CS. We speculated that the application of farm manure increases the soil organic matter and nutrients required for *Mortierella*. However, the interaction between plants and soils may mediate the formation of a specific micro-environment in the rhizosphere soil and generate new dominant populations, which further squeezes the living space of other organisms and results in a decrease of the other microbial populations. Some *Trichoderma* species can release nutrients to promote plant growth and development, and induce plant resistance by activating hormone-mediated (salicylic acid, jasmonic acid, strigolactones, etc.) plant-defence mechanisms [41–43]. *Trichoderma* is usually used as a PGPR in agricultural production to reduce the use of chemical pesticides and fertilizers. *Fusarium* produces culture filtrates toxic to nematodes [44] and can potentially protect plants by suppressing the growth of pathogens and depriving niches [45]. Additionally, it is also a well-known soil-borne phytopathogenic species that can cause severe crop losses [21, 46, 47].

In this study, the cultivation significantly increased both the richness and diversity of soil bacteria compared to those in NCS (Figs 3 and 4). Cultivation mediated the substitution of *Aquabacterium* by *Acidobacteria subgroup-6* as one of the dominant species crucial for the metabolic properties of Fe(II) and nitrate nitrogen in the anaerobic environment. This may be related to soil ploughing, organic fertilizer use and an increase in soil oxygen content [48, 49]. In addition, evident differences were detected in the distribution and abundance of bacterial genera between the rhizosphere and nonrhizosphere. The results revealed that the bacterial species (except for *Haliangum* and *Blii41*) and abundances showed smaller differences between RP and RY in the first 10 genera, and *Bacillus* as the most abundant genus accounted for approximately 40%. Interestingly, *Pseudarthrobacter* only appears in the rhizosphere, which contains a large number of functional genes that show high efficiency in the degradation of phthalic acid esters (PAEs) and help plant roots reduce the absorption of carcinogenic and teratogenic toxic chemicals [50]. Notably, it was also found that *Burkholderia*, acting as a PGPR [51], was abundant in RP, CS, and NCS samples, but only showed a low abundance in RY. This result indicated that the micro-environment produced by the rhizosphere of yellow passion fruit may not be conducive to its growth and reproduction, and further research is needed to explore the cause of this phenomenon.

The plant-associated microbial community is referred to as the second genome of the plant [52], which has been found to successfully suppress plant diseases in previous studies [53]. Plants can interact with the soil and microorganisms to change the soil’s physical and chemical properties by releasing root exudates, such as sugars, amino acids, organic acids, phenolic substances, secondary metabolites and proteins, thus promoting the enrichment of unique rhizosphere microorganisms [54]. It can not only lead to the enrichment of pathogens but also promote the recruitment of beneficial antagonistic bacteria [20]. *Fusarium* and *Trichoderma* were enriched differently between the resistant cultivar (yellow fruit) and the susceptible cultivar (purple fruit) in the same planting environment, and they showed a stronger correlation (Fig 7). Many studies confirmed that *Trichoderma* can antagonize pathogenic bacteria through re-parasitisation, nutritional competition and resistance induction [55–57], and exhibit obvious antagonism to resist *Fusarium graminearum* in maize [58]. According to the results of this study, *Trichoderma* may potentially make huge contributions to sugar metabolism (anaglycolysis-PWY and nonoxipent-PWY), fat metabolism (glyoxylate bypass) and energy metabolism (panto-PWY) (Fig 8). Thus, we inferred that *Trichoderma* may play a major role in the rhizosphere micro-environment of yellow passion fruit, which may be an important reason why yellow fruit plants have stronger resistance to stem rot. Therefore, using *Trichoderma* to control the stem rot of passion fruit may be one of the most economical, effective and environment-friendly methods.

According to previous reports, the invasion of aboveground pathogens can cause changes in long-chain fatty acids, amino acids and malic acids in root exudates, promote the enrichment of potentially beneficial bacteria, such as *Pseudomonas* and *Bacillus subtilis* FB17, and enhance plant resistance [59, 60]. Interestingly, RY and RP originated from healthy plants without disease spots, insect infestations or mechanical damage, and the differences in scions also induced the differential aggregation of *Trichoderma*, *Fusarium* and other microorganisms in the rhizosphere. This result is consistent with Ling’s finding that grafting can shift root exudates and increase the resistance of watermelon plants to *Fusarium*-mediated wilt [61]. However, more studies are needed to explore the aboveground how to regulate the underground microbial community by grafting different scions.

Hence, it can be conceivably hypothesized that there may be some signalling molecules in yellow passion fruit that regulate the production of specific root exudates and exert an obvious promoting effect on *Trichoderma* or inhibit the growth of other microorganisms to increase the ecological space. Comparative genomics combined with metabolomics can be adopted to explore the functional genes of specific secretions released by plants, cultivate varieties with high resistance to stem rot, and separate the specific secretions. This may contribute to the development of biological pesticides and biofertilizers, which can recruit *Trichoderma* to prevent and control the growth of phytopathogenic bacteria.

## Conclusion

Continuous cropping of passion fruit alters the richness and diversity of soil bacterial and fungal communities. Differential aggregation of rhizosphere microbial communities may result from the grafting of different scions and may lead to the formation of a unique rhizosphere microorganism environment under continuous cropping. The massive accumulation of beneficial antagonistic fungi in the rhizosphere, such as those belonging to the genus *Trichoderma*, may be an important source of stronger disease resistance in yellow passion fruit. Therefore, exploring the factors and regulatory mechanisms that cause *Trichoderma* enrichment in the rhizosphere will be beneficial to biological control, pesticide reduction and quality improvement.

## Supporting information

S1 Table Sequencing statistics of different soil and rhizosphere samples for fungi and bacteria. A) Amount of sequences that can be matched to both the forward and reverse primers. (B, C and D) Number of sequences after removing, denoising and splicing. (E and F) Number of sequences which the chimeras and singletons were removed.
(XLS)

S2 Table The relative abundance of *Mortierellomycota* in different soil samples. (XLS)

S3 Table The numbers of 16S rRNA ASVs annotated to the family and genus level. (XLS)

S4 Table The abundance of *Firmicutes* and *Acidobacteria* in different samples. (XLS)

S5 Table The abundance of *Subgroup_2*, *AD3* and *Bacillus* in different samples. (XLSX)

S6 Table The analysis of different metabolic pathways between RP and NCS for fungi. (XLS)

S7 Table The analysis of different metabolic pathways between RY and NCS for fungi. (XLS)

S8 Table The analysis of different metabolic pathways between CS and NCS for fungi. (XLS)

S9 Table The analysis of different metabolic pathways between RP and NCS for bacteria. (XLS)

S10 Table The analysis of different metabolic pathways between RY and NCS for bacteria. (XLS)

S11 Table The analysis of different metabolic pathways between CS and NCS for bacteria. (XLS)

S1 Fig Analysis of overall secondary function for fungi (a) and bacteria (b). The abscissa and ordinate represent the average abundance and different functional pathways at the second level and the first level of pathway classification is shown on the right side.
(TIF)
