# Supplementary figures and images for: Passion fruit plants alter the soil microbial community with continuous cropping and improve plant disease resistance by recruiting beneficial microorganisms
Source: PLoS One. 2023 Feb 21;18(2):e0281854. doi: 10.1371/journal.pone.0281854 (PMC9943001; doi:10.1371/journal.pone.0281854)

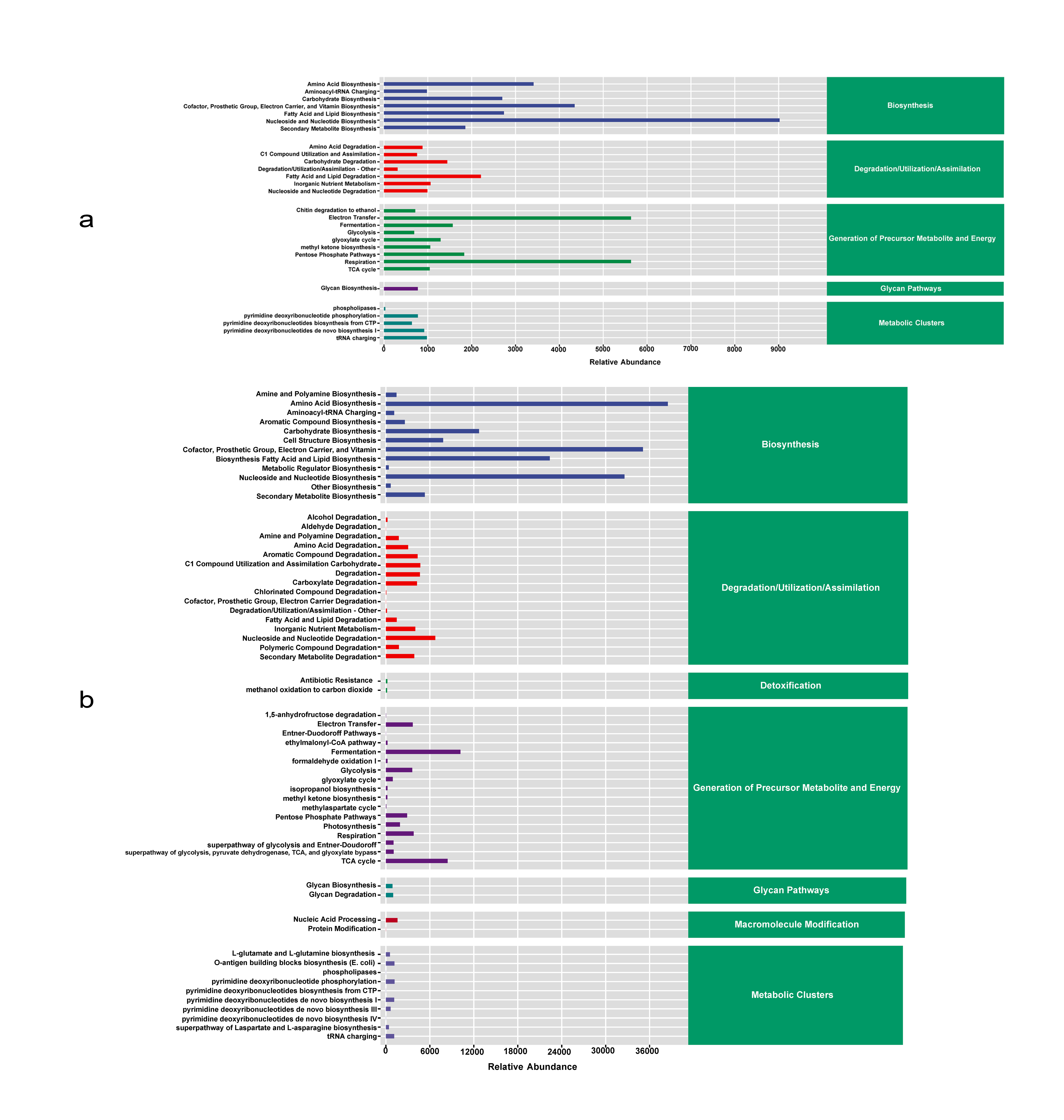

Supplement: S1 Fig — Analysis of overall secondary function for fungi (a) and bacteria (b). The abscissa and ordinate represent the average abundance and different functional pathways at the second level and the first level of pathway classification is shown on the right side. (TIF) [file pone.0281854.s012.tif]
